# Editorial: Insights Into the Etiology, Prevention, and Treatment of Food Allergy

**DOI:** 10.3389/fimmu.2020.01937

**Published:** 2020-08-21

**Authors:** Michiko Oyoshi, R. Sharon Chinthrajah

**Affiliations:** ^1^Boston Children's Hospital, Harvard Medical School, Boston, MA, United States; ^2^Sean N. Parker Center for Allergy and Asthma Research, Stanford University, Stanford, CA, United States

**Keywords:** food allergy, immunology, allergic diseases, immunotherapy, food hypersensitivity

Food allergy (FA) is a growing public health concern affecting nearly up to 6–8% in children and 3–5% in adults with life-threatening potential. The increase in prevalence of FA in recent years has implicated environmental influences related to a modern life style in disease pathogenesis. This Frontiers Research Topic was designed to provide a timely collection on animal and clinical studies on new research directions in the etiology, prevention, and treatment of FA. We received a series of 14 original and review articles on the topic that span a wide range of exciting, new areas that will inform the research community on FA.

## Etiology and Prevention

FA can be defined as clinical immune responses to normally harmless food allergens. The disease is typically associated with CD4^+^ T cells that secrete pathogenic T helper (Th) 2 cytokines and by allergen-specific immunoglobulin (Ig) E antibodies that trigger the release of inflammatory mediators from mast cells (MCs) and circulating basophils. Normally, ingestion of foods results in oral tolerance, therefore FA is thought to be a result of a failure of oral tolerance. Alterations in regulatory T cell functions, Th2 responses, microbiota, and/or food sensitizations via alternative routes, such as the skin, likely contribute to the failure of oral tolerance and to the development of FA. However, the mechanisms responsible for breakdown in oral tolerance remain poorly understood.

A series of articles were directed at the immune regulatory mechanisms in FA tolerance. Satitsuksanoa et al. reviewed the range of immune regulatory mechanisms of tolerance to FA. Ingested food proteins may cause allergic immune responses leading to FA but how these proteins become immunogenic and cause food allergies is not completely understood. Tolerance to food is mainly acquired by dendritic cells, epithelial cells in the gut, and the gut microbiome. These authors found that a subset of CD103+ DCs is capable of inducing T regulatory cells (Treg cells) that express anti-inflammatory cytokines. Anergic T cells also contribute to oral tolerance by reducing effector cells. Similar to Treg cells, regulatory B cells (Breg cells) suppress effector T cells and contribute to the immune tolerance to food allergens. Saunders et al. reviewed allergic IgE responses and found that IgE memory response has unique features that distinguish it from classical B cell memory. Burton et al. investigated tissue-specific expression of FcγRIIb, a low-affinity IgG receptor on MCs in allergy. The authors combined flow cytometry, quantitative PCR, and immunofluorescence staining of MCs derived from the tissues of humanized mice, human skin, or fixed paraffin-embedded sections of human tissues, and demonstrated that FcγRIIb is absent from dermal MCs but expressed by MCs throughout the gastrointestinal tract. IgE-induced systemic anaphylaxis in humanized mice is strongly inhibited by antigen specific IgG; thus, these authors concluded that IgG signaling via FcγRIIb plays a role in suppressing hypersensitivity reactions. Krajewski et al. found that epigenetic regulation of MC activation during immune responses may occur via altered histone acetylation, and that exposure to dietary substances may induce epigenetic modifications that regulate MC function.

The skin is a major immunologic organ that may induce protection, sensitization, or tolerance. Epicutaneous immunotherapy (EPIT) has been proposed as an attractive strategy to actively treat FA in humans. Dioszeghy et al. demonstrates that EPIT induced tolerance in sensitized mice through the induction of Foxp3+ regulatory T cells (Tregs), especially CD62L+ Tregs. Although both Langerhans cells (LCs) and CD11b+ dermal classical dendritic cells could induce Tregs, the absence of LCs during EPIT impaired treatment efficacy, indicating that LCs play a crucial role in skin-induced tolerance.

The regulation of gut antibody responses also plays a role in regulating food allergens. In a review, Hoh and Boyd showed that the gastrointestinal mucosa is a critical environmental interface in which plasma cells and B cells are exposed to orally-ingested antigens such as food allergen proteins. It is unclear how the development of B cells and plasma cells in the gastrointestinal mucosa differs between healthy humans and those with FA, and how B cells contribute to, or are affected by, the breakdown of oral tolerance. Furthermore, the human microbiome is an essential mediator in the induction of oral tolerance or FA. In another study with germ-free mice, Schwarzer et al. showed that microbiota-induced maturation and gut-homing of MCs is a critical step for the development of symptoms of experimental FA. This new mechanistic insight into microbiota-MC-FA axis could be exploited in preventing and treating FA in humans. Hayen et al. found that intestinal epithelial exposure to short-chain galacto-oligosaccharides and long-chain fructo-oligosaccharides enhanced the CpG induced Th1 and regulatory IL-10 response in a peanut-specific co-culture model. This suggests that such oligosaccharides are candidate for dietary adjunct in allergen-specific immunotherapy.

While oral immunotherapy (OIT) has shown promise in treating food allergies, gastrointestinal symptoms are experienced by patients and few develop eosinophilic gastrointestinal disease. Wright et al. found pre-existing gastrointestinal eosinophilia is common in adults with IgE-mediated peanut allergy. Eosinophilic inflammation in these subjects may be accompanied by mild endoscopic and histologic findings. Further, Sallis et al. found that eosinophilic esophagitis (EoE) patients with food impaction were indistinguishable from other EoE patients based on their tissue eosinophil count, serum IgE levels, or the mRNA transcriptome-based a probability score for EoE [p(EoE)] but in an elegant analysis, they showed that a distinct esophageal mRNA pattern identified EoE patients with food impactions. The EoE-specific mRNA pattern indicates that impaired motility may be one underlying factor for the development of food impactions in pediatric patients.

Finally, Fujimura et al. reviewed the influences of maternal factors over offspring allergies. They reviewed how food allergens, allergen-specific immunoglobulins, cytokines, genetics, and environmental factors transferred during pregnancy or breastfeeding influence offspring allergies and how such effects may be applicable to FA. They also discussed the mechanisms by which maternal factors, including the impact of immune complexes, transforming growth factor-β, vitamin A, and regulatory T-cell responses, contribute to the induction of neonatal tolerance vs. development of allergic responses to maternally transferred allergens.

## Treatment

There is currently no cure for FA other than strict avoidance of identified foods. Key problems in this field that remain to be resolved are our insufficient understanding of the mechanisms of the breakdown in oral tolerance in FA and our understanding of the reasons why such mechanisms have recently taken such a strong base in the human population. We are at an exciting point at which discoveries about the etiology, mechanisms, treatment, and prevention of FA are critical to guiding future areas of research and identifying therapeutic options for food allergic patients.

Currently, two forms of peanut immunotherapy, OIT and EPIT, are in Phase III clinical trials and have shown promise to desensitize patients with FA. However, there are several limitations with OIT and EPIT, such as allergic side effects, daily dosing requirements, and the infrequent outcome of long-term tolerance. Next-generation therapies for peanut allergy may overcome these limitations, particularly with adjuvanted immunotherapy. Johnson-Weaver et al. reviewed adjuvants and formulations that have shown pre-clinical efficacy in treating peanut allergies. In an analysis of 428 participants with positive food challenges, Purington et al. found those with a history of asthma, high allergen-specific IgE to total IgE ratio, and/or high values of allergen-specific IgE were also found to be at higher risk for severe reactions during food challenges. Their findings will help optimize food challenge dosing schemes in multi-food allergic, atopic patients, specifically at lower doses where the majority of reactions occur. In an analysis of a large food challenge dataset, baseline population characteristics, biomarkers, and challenge outcomes were studied to develop diagnostic criteria predictive of positive oral food challenges (OFCs) across multiple allergens in our multi-allergic cohorts (Sindher et al.). They found a history of atopic dermatitis and multiple food allergies were significantly associated with a higher risk of positive OFCs. The majority of food-specific skin prick tests, allergen-specific IgE, and allergen-specific IgE/total IgE thresholds calculated from cumulative tolerated dose (CTD)-dependent receiver operator curves had high discrimination of OFC outcomes and participants with values above the thresholds were more likely to have positive challenges.

Overall, we strove to provide an overview of recent progress in the field of food allergy. Our 14 research articles provided novel findings in the field of environmental and genetic risk factors for food allergy, the mechanisms of food allergy: effector immune cells involved in the initiation, development and manifestations of disease, regulatory immune mechanisms, and insights into the prevention and treatment of food allergy.

## Author Contributions

RC and MO: wrote and contributed substantial intellectual contribution to this editorial.

## Conflict of Interest

The authors declare that the research was conducted in the absence of any commercial or financial relationships that could be construed as a potential conflict of interest.

